# Supplementary material for: Predicting visual function by interpreting a neuronal wiring diagram
Source: Nature. 2024 Oct 2;634(8032):113–23. doi: 10.1038/s41586-024-07953-5 (PMC11446822; doi:10.1038/s41586-024-07953-5)
Supplement: Supplementary file 6 — CRF and ERF predictions for individual TmY4 and TmY9 cells. Analogous to Supplementary Data 3, but for TmY target types. Shown are the top four monosynaptic pathways, the strongest pathway passing through each of the top ten intermediary types (ranking from Extended Data Fig. 7), and the trisynaptic pathway Tm1–TmY–Dm3–TmY (see the section entitled Prediction of spatial normalization). [file 41586_2024_7953_MOESM6_ESM.zip › DataS4/TmY9qΓèÑ/720575940613443746.pdf]

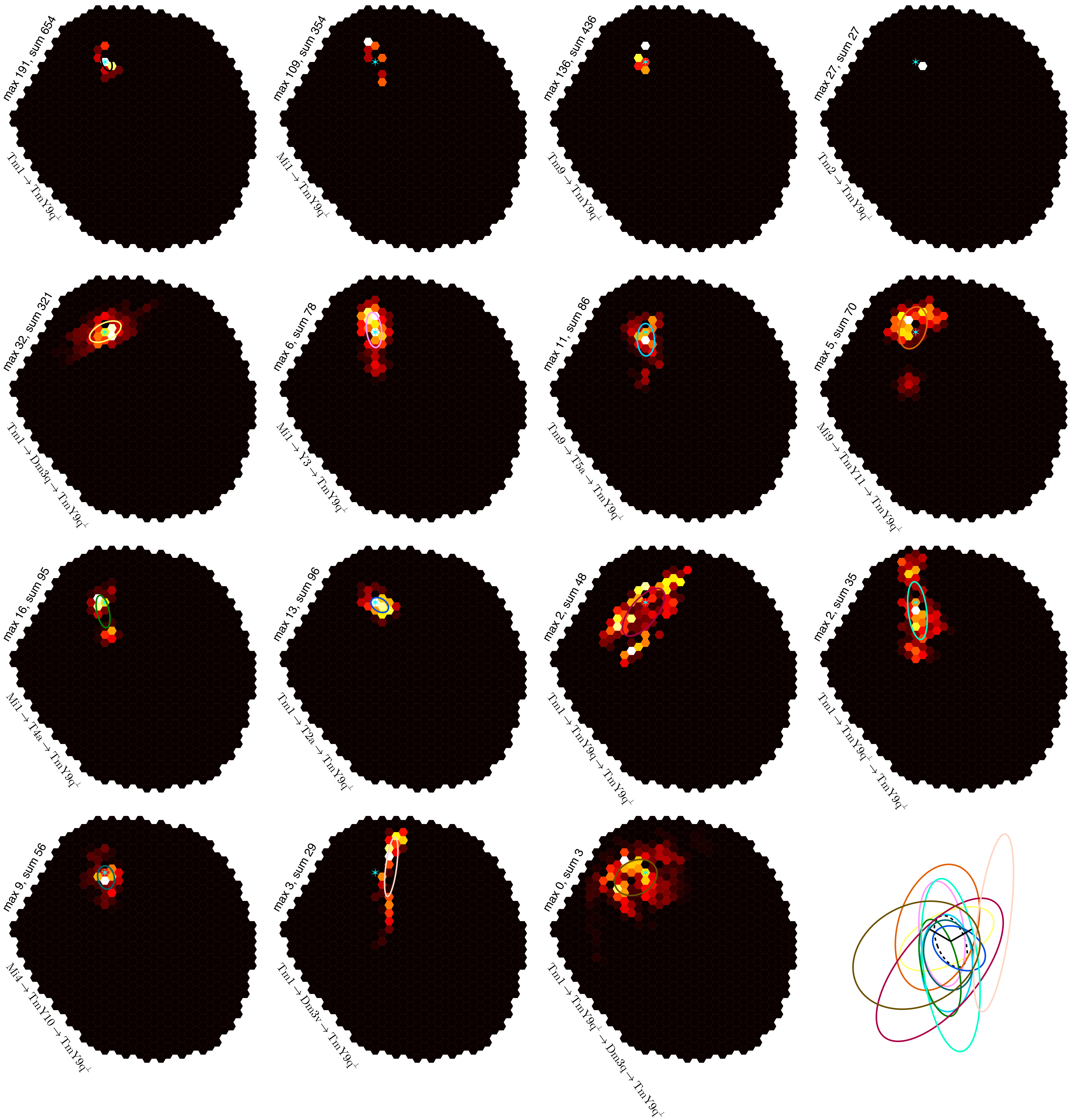
max 191, sum 654
Tm1 → TmY9q⊥
max 109, sum 354
Mi1 → TmY9q⊥
max 136, sum 436
Tm9 → TmY9q⊥
max 27, sum 27
Tm2 → TmY9q⊥
max 32, sum 321
Tm1 → Dm3q → TmY9q⊥
max 6, sum 78
Mi1 → Y3 → TmY9q⊥
max 11, sum 86
Tm9 → T5a → TmY9q⊥
max 5, sum 70
Mi9 → TmY11 → TmY9q⊥
max 16, sum 95
Mi1 → T4a → TmY9q⊥
max 13, sum 96
Tm1 → T2a → TmY9q⊥
max 2, sum 48
Tm1 → TmY9q → TmY9q⊥
max 2, sum 35
Tm1 → TmY9q⊥ → TmY9q⊥
max 9, sum 56
Mi4 → TmY10 → TmY9q⊥
max 3, sum 29
Tm1 → Dm3v → TmY9q⊥
max 0, sum 3
Tm1 → TmY9q⊥ → Dm3q → TmY9q⊥
